# A gastric invasive tubular adenocarcinomatous lesion arising from foveolar-type neoplasia: molecular histogenesis

**DOI:** 10.1007/s12328-024-01974-3

**Published:** 2024-04-25

**Authors:** Tamotsu Sugai, Noriyuki Uesugi, Koichi Hamada, Takayuki Nagahashi, Yoshinori Horikawa, Masamichi Suzuki, Ryo Sugimoto, Naoki Yanagawa

**Affiliations:** 1 Diagnostic Pathology Center, Southern Tohoku General Hospital, 7-115 Yatsuyamada, Koriyama, Fukushima 963-8563 Japan; 2 Department of Molecular Diagnostic Pathology, School of Medicine, Iwate Medical University, 2-1-1 Idaidori, Yahaba, Shiwa, Iwate 028-3695 Japan; 3 Department of Gastroenterology, Southern Tohoku General Hospital, 7-115 Yatsuyamada, Koriyama, Fukushima 963-8563 Japan

**Keywords:** Allelic imbalance, Foveolar-type neoplasia, Molecular analysis, Progression, Tubular differentiated adenocarcinoma

## Abstract

Here, we report a rare case of a depressed lesion exhibiting both tubular differentiated adenocarcinomatous (TDA) and intraepithelial foveolar neoplasia (IFN) components (with the histological appearance of foveolar hyperplasia due to low-grade atypia). Histologically, the TDA surrounded the IFN, suggesting that the TDA may have originated from the IFN. Therefore, we examined molecular alterations in the TDA and IFN components separately. MUC5AC and MUC6 expression was observed immunohistochemically in both components. p53 expression was wild type in both components, suggesting no mutation of *TP53*. We investigated allelic imbalances at multiple loci (1p, 3p, 4p, 5q, 8q, 9p, 13q, *TP53*, 18q, and 22q), mutations (*KRAS*, *BRAF*, and *GNAS*), and DNA methylation and microsatellite status in both components using PCR-based analyses. Although multiple allelic imbalances were common to both components, allelic imbalances at 3p and *TP53* were found only in the TDA component. No mutations were found, and DNA methylation status was low epigenotype for both components. Ultimately, this tumor was considered microsatellite stable. Considering the origin of TDA, which is frequently encountered in routine practice, IFN may develop into TDA.

## Introduction

Gastric cancer (GC) is a major cause of cancer-related death in Japan [1]. The histogenesis of GC has been widely investigated [2–4]. Although two histological types, intestinal and diffuse, have been identified, the histogenesis of GC has not been completely elucidated [5, 6]. The histological classification suggests that GC is heterogeneous [1, 3], and such heterogeneity is found even in early-stage GC [4, 6].

Foveolar-type neoplasia, characterized by similarity to foveolar epithelium, small nuclei in the basal layer, irregular branching, and hyperchromatin, is an uncommon histology of GC [7, 8]. It is difficult, even for expert gastrointestinal pathologists, to differentiate benign from malignant foveolar-type neoplasia [7, 8]. The histological type into which foveolar-type neoplasia differentiates remains unknown [8].

We report an unusual case of histogenesis of early-stage GC in which foveolar-type neoplasia was involved focally and demonstrate its histogenesis at the molecular level.

### Clinical summary

**Fig. 1:**
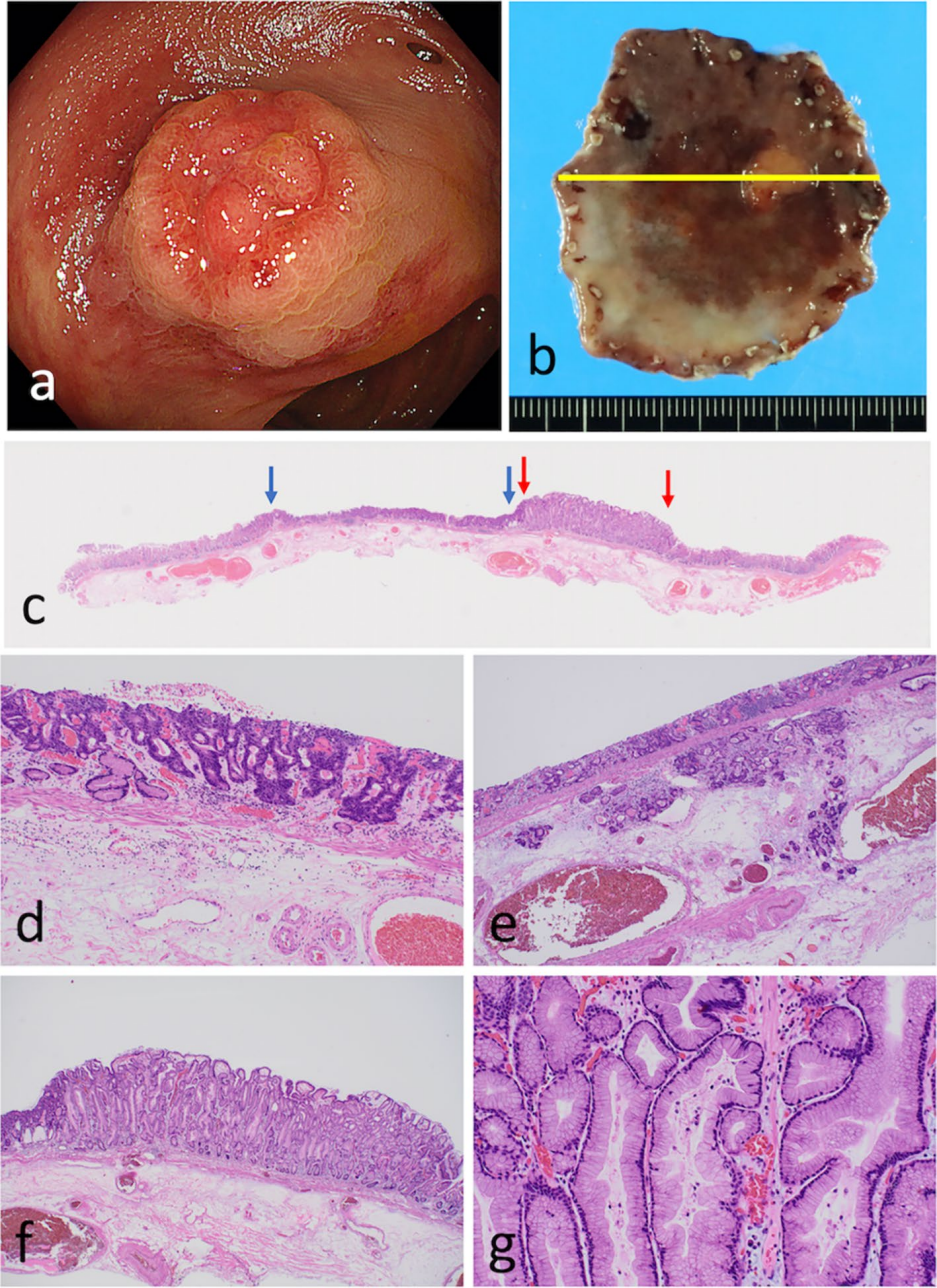
Endoscopic, macroscopic, and histological features of the resected specimen. **a** Endoscopic findings of the lesion. Note the elevated lesion within the depressed lesion. **b** Macroscopic features of the resected specimen. The resected specimen was cut at 3 mm intervals. The specimen consisted of a slightly depressed lesion measuring 43 mm × 23 mm and a focally elevated lesion measuring 12 mm × 11 mm within the slightly depressed lesion. The bold yellow lines denote representative sections. The histology of the section is indicated by the bold yellow lines in panel **c** A representative loupe histological photograph of the resected specimen (hematoxylin and eosin staining; × 2; depressed lesion, blue arrow; elevated lesion, red arrow). **d** Low-power view of a hematoxylin and eosin-stained section showing irregular and complex glands with highly pleomorphic nuclei. **e** Lymphatic invasion is observed in the submucosal layer. **f** Low-power view of a hyperplastic-like elongated gland with low-grade atypia located at the basal membrane. **g** High-power view of the histological section showing cells with low N/C and hyperchromatin

A 55-year-old man with a previous diagnosis of colonic adenoma, hypertension, and diabetes mellitus visited Southern Tohoku General Hospital due to a positive result for fecal occult blood. Although he had no physical symptoms when visiting the hospital, he underwent an endoscopy for further examination. His laboratory test results were within the normal range. Upper gastrointestinal tract endoscopy revealed a reddish depressed lesion with a focal polypoid area in the posterior wall of the antrum (Fig. 1a). Although the endoscopic diagnosis for the depressed area was intramucosal adenocarcinoma, the association of the endoscopic findings between the depressed area and polypoid lesion was unclear. Endoscopic submucosal dissection was performed based on the endoscopy findings. A serum antibody test for *Helicobacter pylori* was performed. The antibody test was negative at the time of consultation due to past *H. pylori* eradication therapy performed in 2018. Endoscopy was not conducted from 2018 to 2023.

### Pathological findings

The endoscopic submucosal dissection specimen consisted of two histological features corresponding to depressed and elevated lesions, measuring 43 mm × 23 mm (Fig. 1b). The depressed lesion comprised irregularly branched neoplastic glands with high nuclear pleomorphism and mitotic activity (Fig. 1c, d). The neoplastic glands had invaded the submucosa, where lymphatic invasion was found (Fig. 1e). The lesion was considered a tubular differentiated adenocarcinoma (conventional adenocarcinoma). A hyperplastic-like lesion revealing a sessile elevated lesion macroscopically was observed within the elevated lesion (Fig. 1f, g). This hyperplastic-like lesion demonstrated elongated glands with mild nuclear pleomorphism and no mitotic activity (Fig. 1g); however, hyperchromatin was found in the nuclei (Fig. 1g). The transitional area may help determine the origin of the two histological components. If the transitional area between the two components was observed in the lesion, the lesion was considered to be the sole origin. However, a transitional area between the two components was not clearly observed in the present case. The differential diagnosis includes hyperplastic lesions and foveolar-type neoplasia. Although no nuclear pleomorphism was observed in the cells, we diagnosed the hyperplastic-like lesion as low-grade intraepithelial foveolar neoplasia (IFN) due to the presence of irregular branching of neoplastic glands and hyperchromatic nuclei. Ultimately, atrophic gastric mucosa with intestinal metaplasia (histopathologically, chronic gastritis) was observed in the background mucosa of the lesion.

### Molecular findings

**Fig. 2:**
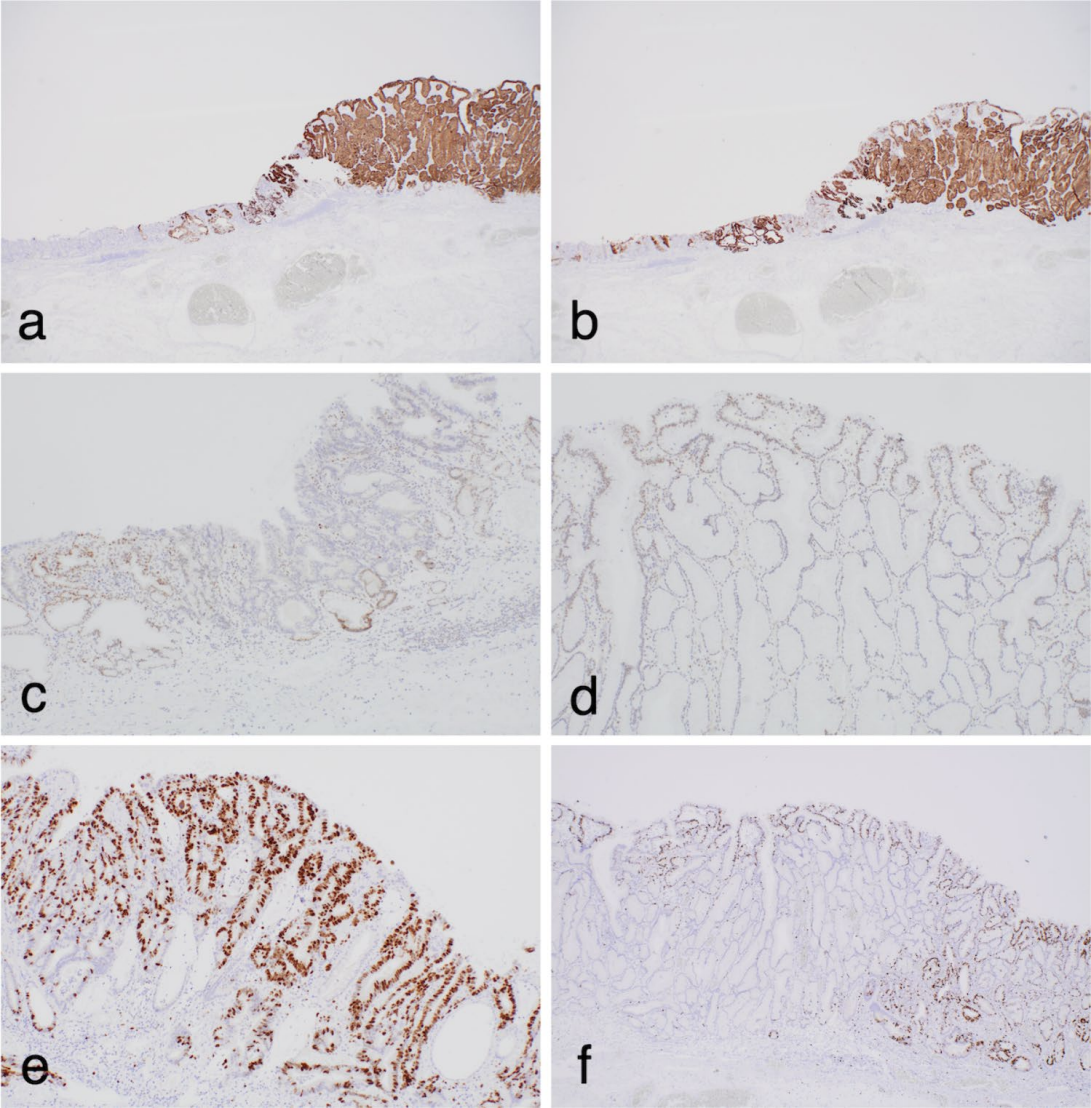
Immunohistochemistry. **a** Immunoreactivity of MUC5AC in both components. **b** Immunoreactivity of MUC6 in both components. **c**, **d** Immunoreactivity of p53 in both components. **e** Immunoreactivity of Ki-67 in the depressed component. **f** Immunoreactivity of Ki-67 in the elevated component

Immunohistochemical staining (Dako Envision) revealed positive immunoreactivity for MUC5AC and MUC6 but negative for MUC2 (Fig. 2a, b), suggesting a gastric mucin phenotype. Although weak p53 immunoreactive expression was detected in the tubular differentiated adenocarcinomatous (TDA) and IFN components, it was considered the wild type (Fig. 2c, d). By contrast, expression of Ki-67 was significantly greater in the TDA (76.6%) than in the IFN (4.3%) component (Fig. 2e, f).

The TDA and IFN components were microdissected separately for molecular analysis (Table 1). We examined allelic imbalances (AIs), which are closely associated with irreversible genomic changes. The allelic loci examined were 1p, 3p, 4p, 5q, 8q, 9p, 13q, *TP53*, 18q, and 22q, which are frequently affected in GC [6]. The microsatellite sequences were amplified by PCR using specific primers obtained from the Genome Database (http://gdbwww.gdb.org/gdb/), and a thermal cycler (GeneAmp PCR System 9600; PerkinElmer) was used as described previously [6]. If the expression of at least one of the multiple markers examined within a chromosomal locus was classified as positive, the AI status of that locus was considered positive. Although multiple AIs were commonly detected in both components, AIs at 3p and *TP53* were detected in the TDA but not in the IFN component (Table 1). The allelic loci showing the AIs that we tested were the same loci between the two components. In addition, the MSI status was analyzed in a similar way [9]. MSI status was determined by 5 NCI markers, namely, BAT25, BAT26, D2S123, D5S346, and D17S250. MSI-high (MSI-H) was defined as two or more markers being unstable, MSI-low (MSI-L) was defined as 1 marker being unstable, and microsatellite stable (MSS) was defined as the absence of marker instability [9]. The present patient had MSS in both components. Mutations in *KRAS*, *BRAF,* and *GNAS* were quantified by PCR analysis of bisulfite-modified genomic DNA (EpiTect Bisulfite Kit; Qiagen) using pyrosequencing (PyroMark Q24; Qiagen), as previously described [10]. No mutations were detected in either component (Table 1). Finally, PCR-based DNA methylation analysis was performed following published protocols [11]. In brief, DNA methylation at the promoter regions of six genes was quantified (PyroMark Q24; Qiagen). The cutoff value for methylation status was determined to be 15%. Tumors with methylation of at least two of three markers (RUNX3, MINT31, and LOX) were defined as having a highly methylated epigenotype (HME). The remaining tumors without HME were screened for methylation of three other markers (NEUROG1, ELMO1, and THBD) and were defined as having an intermediate methylation epigenotype (IME) if at least two of these markers were methylated. Tumors not classified as HME or IME were defined as having a low methylation epigenotype (LME). DNA methylation analysis revealed an LME in both components. The results of the molecular analyses are shown in Table 1. In conclusion, we suggest that histologically, TDA originates from the IFN.

**Table 1:** Molecular analyses of the tubular differentiated adenocarcinomatous and intraepithelial foveolar intraepithelial neoplasia components

	TDA component	IFN component
Allelic imbalance		
1p	positive	positive
3p	positive	negative
4p	positive	positive
5q	positive	positive
8q	positive	positive
9p	positive	positive
13q	positive	positive
* TP53*	positive	negative
18q	positive	positive
22q	positive	positive
Gene Mutation		
* KRAS*	negative	negative
* BRAF*	negative	negative
* GNAS*	negative	negative
MSI	negative	negative
DNA methylation	LME	LME

*TDA* tubular differentiated adenocarcinomatous, *IFN* intraepithelial foveolar neoplasia, *MSI* microsatellite instability, *LME* low-methylation epigenotype

## Discussion

In the present case, TDA and IFN components (with the histological appearance of foveolar hyperplasia; Fig. 1f) were observed within the same tumor. However, whether the TDA component originated from the IFN component, as a precursor lesion is unclear; this histological progression from IFN may be missed, leading to misdiagnosis of the present case as conventional adenocarcinoma (known as tubular and papillary adenocarcinoma in Japan).

We suggest that early GC (an invasive carcinoma involving only the stomach mucosa or submucosa, independent of lymph node status) can be primarily classified into two molecular phenotypes, genetic- and epigenetic-dominant types, based on previous studies [4, 12]. The epigenetic-dominant type is characterized by high DNA methylation and few genomic changes during early GC development. By contrast, the genetic-dominant type exhibits an accumulation of genetic changes (i.e., chromosomal instability) in early GC [2, 4]. According to molecular data, the present case is considered the genetic-dominant type distinct from the epigenetic-dominant type seen in conventional adenocarcinoma. However, the molecular classification of an individual gastric adenocarcinoma is not clear, with rare tumors comprising a mixture of conventional and foveolar features.

*TP53* mutations may activate the proliferation of cancer cells and play an important role in gastric carcinogenesis [2, 6, 13]. In the present study, the p53 immunostaining pattern in both lesions showed a wild-type pattern, suggesting the presence of wild-type *TP53* in both lesions. This finding suggested that a *TP53* mutation-dependent pathway plays no fundamental role in either lesion. Regardless of the p53 immunophenotype, greater proliferative activity was observed in the TDA than in the IFN component. Multiple AIs contribute to increased proliferation [6]; however, AIs were unlikely to be responsible for the high proliferative activity in the TDA, given that the same AIs were also present in the IFN component. Unfortunately, it is unclear why the proliferative activity was greater in the TDA than in the IFN component.

Whether IFN is benign or malignant is controversial [7, 8]. A recent study showed that multiple AIs are detected in gastric IFN, suggesting that despite low-grade atypia, IFN has a malignant nature [8]. This finding implies that TDA does not resemble IFN as an invasive phenotype of IFN. Although whole exome sequencing (WES) may ideally be required to prove the clonal identity of both lesions, such a method is unsuitable for paraffin-embedded tissue. In addition, the WES method is more expensive and time-consuming than AI analysis. Multiple AI analyses are helpful in identifying associations among different histological types, given that the AI pattern of conventional tubular adenocarcinoma is distinct from that of IFN [6, 8].

In conclusion, we report a rare case of early GC with natural disease progression from IFN with low-grade atypia to high-grade TDA, both pathologically and molecularly. To our knowledge, this is the first report of IFN (with the histological appearance of foveolar hyperplasia) progressing into a high-grade TDA lesion. Furthermore, this case is unique in that a high-grade TDA component with high proliferative activity replaced an IFN component, according to molecular alterations. Further analysis of similar cases is needed to discover a new pathway involved in the progression from IFN to elucidate the clinicopathological behavior of IFN.

## References

[1] Sitarz R, Skierucha M, Mielko J, et al. Gastric cancer: epidemiology, prevention, classification, and treatment. Cancer Manag Res. 2018;10:239–48. 29445300 10.2147/CMAR.S149619 PMC5808709

[2] Cancer Genome Atlas Research Network. Comprehensive molecular characterization of gastric adenocarcinoma. Nature. 2014;513:202–9. 25079317 10.1038/nature13480 PMC4170219

[3] Kushima R, Lauwers GY, Rugge M. Gastric dysplasia: WHO classification of tumours of the digestive system. Lyon: International Agency for Research on Cancer; 2019. pp. 71–75.

[4] Sugai T, Inomata M, Uesugi N, et al. Analysis of mucin, p53 protein and Ki-67 expressions in gastric differentiated-type intramucosal neoplastic lesions obtained from endoscopic mucosal resection samples: a proposal for a new classification of intramucosal neoplastic lesions based on nuclear atypia. Pathol Int. 2004;54:425–35. 15144402 10.1111/j.1440-1827.2004.01643.x

[5] Zheng H, Takahashi H, Murai Y, et al. Pathobiological characteristics of intestinal and diffuse-type gastric carcinoma in Japan: an immunostaining study on the tissue microarray. J Clin Pathol. 2007;60:273–7. 16714395 10.1136/jcp.2006.038778 PMC1860577

[6] Sugai T, Sugimoto R, Habano W, et al. Genetic differences stratified by PCR-based microsatellite analysis in gastric intramucosal neoplasia. Gastric Cancer. 2017;20:286–96. 27236438 10.1007/s10120-016-0616-2

[7] Sekine S, Montgomery EA, Vieth M. Foveolar type adenoma: WHO classification of tumours of the digestive system. Lyon: International Agency for Research on Cancer; 2019. pp. 79–80.

[8] Sugai T, Uesugi N, Habano W, et al. The clinicopathological and molecular features of sporadic gastric foveolar type neoplasia. Virchows Arch. 2020;477:835–44. 32533343 10.1007/s00428-020-02846-0 PMC7683467

[9] Boland CR, Thibodeau SN, Hamilton SR, et al. A National Cancer Institute Workshop on Microsatellite Instability for cancer detection and familial predisposition: development of international criteria for the determination of microsatellite instability in colorectal cancer. Cancer Res. 1998;58:5248–57. 9823339

[10] Sugai T, Eizuka M, Takahashi Y, et al. Molecular subtypes of colorectal cancers determined by PCR-based analysis. Cancer Sci. 2017;108(3):427–34. 10.1111/cas.13164. 28083970 10.1111/cas.13164 PMC5378279

[11] Yagi K, Takahashi H, Akagi K, et al. Intermediate methylation epigenotype and its correlation to KRAS mutation in conventional colorectal adenoma. Am J Pathol. 2012;180:616–25. 22115708 10.1016/j.ajpath.2011.10.010

[12] Arakawa N, Sugai T, Habano W, et al. Genome-wide analysis of DNA copy number alterations in early and advanced gastric cancers. Mol Carcinog. 2017;56:527–37. 27312513 10.1002/mc.22514

[13] Skierucha M, Milne AN, Offerhaus GJ, et al. Molecular alterations in gastric cancer with special reference to the early-onset subtype. World J Gastroenterol. 2019;22:2460–74. 10.3748/wjg.v22.i8.2460 PMC4768192 26937134

